# A preliminary exploration of the regression equation for performance in amateur half-marathon runners: a perspective based on respiratory muscle function

**DOI:** 10.3389/fphys.2024.1340513

**Published:** 2024-03-25

**Authors:** Houyuan Zhu, Xiaowei Han, Guoqing Miao, Qi Yan

**Affiliations:** ^1^ China Institute of Sport Science, Beijing, China; ^2^ School of Physical Education, Hebei Normal University, Shijiazhuang, Hebei, China

**Keywords:** half marathon, respiratory muscle, maximum expiratory pressure, multiple linear regression, running

## Abstract

This document presents a study on the relationship between physical characteristics, respiratory muscle capacity, and performance in amateur half-marathon runners. The aim of this study was to establish a preliminary predictive model to provide insights into training and health management for runners. Participants were recruited from the 2023 Beijing Olympic Forest Park Half-Marathon, comprising 233 individuals. Personal information including age, gender, height, weight, and other relevant factors were collected, and standardized testing methods were used to measure various parameters. Correlation analysis revealed significant associations between gender, height, weight, maximum expiratory pressure, maximal inspiratory pressure, and half-marathon performance. Several regression equations were developed to estimate the performance of amateur marathon runners, with a focus on gender, weight, maximum expiratory pressure, and height as predictive factors. The study found that respiratory muscle training can delay muscle fatigue and improve athletic performance. Evaluating the level of respiratory muscle capacity in marathon athletes is crucial for defining the potential speed limitations and achieving optimal performance. The information from this study can assist amateur runners in optimizing their training methods and maintaining their physical wellbeing.

## 1 Introduction

With the changing modern lifestyle and the heightened health consciousness among individuals, an increasing number of people are actively engaging in running activities, considering it as one of the means to promote physical fitness and derive enjoyment from life. As a highly popular long-distance running event, the half marathon has garnered significant participation from amateur runners (Anthony et al., 2014; Knechtle et al., 2016; Divine et al., 2018; Cuk et al., 2020). For these amateur runners, the pursuit of running extends beyond the pleasure it brings; they also seek to gain insights into their physical condition and athletic prowess, aiming to optimize their training methods and maintain optimal physical wellbeing (Hammer and Podlog, 2016; Zach et al., 2017).

The marathons is long-distance running races that cover a distance of 42.195 km. Since the first modern Olympic Games held in Athens in 1896 to the inaugural “city marathon” in New York City in 1976 (Burfoot, 2007), the popularity of marathon events has grown steadily, transforming into a global social phenomenon (Vitti et al., 2020). Among them, the half marathon, as a challenging yet relatively shorter marathon race, has also gained popularity among running enthusiasts (Nikolaidis et al., 2019). The number of participants in half marathons has been increasing annually in Europe and America (Anthony et al., 2014; Knechtle et al., 2016), while in China, there has been a rise in marathon events that include a half marathon category (Zuo et al., 2019). As the enthusiasm for these events continues to grow (Burfoot, 2007; Knechtle et al., 2018; Vitti et al., 2020)], the range of participants has expanded, accommodating both amateur and elite runners, and the performance of participants has steadily improved (Gómez-Molina et al., 2017). Marathon events are influenced by various uncontrollable factors for runners, such as climate conditions including temperature, humidity, and atmospheric pressure, which exhibit seasonal characteristics (Weiss et al., 2022a; Weiss et al., 2022b). Individual characteristics such as age, gender, physical fitness, psychological traits, and conditions, as well as training variables including tactics and pacing strategies, also play a significant role (Boullosa et al., 2020; Cuk et al., 2021). For instance, regarding environmental factors (Weiss et al., 2022a), temperature and humidity have different effects on the pacing of marathon runners in different age groups. In terms of body measurements, some studies have found a negative correlation between half marathon times and body weight (Hoffman, 2008; Knechtie et al., 2009; Zillmann et al., 2013). Regarding training variables, performance of athletes is positively correlated with certain training variables, such as weekly running distance (in kilometers), weekly training frequency, average exercise speed, and weekly training hours (Rüst et al., 2011). Overall, males tend to be faster than females (Vitti et al., 2020), experienced runners have higher step frequencies and lower energy expenditure compared to novices (De Ruiter et al., 2014), and better athletic performance can be achieved through shorter ground contact times (Santos-Concejero et al., 2013).

During long-distance running, the function of the respiratory muscles plays a crucial role in athletes’ endurance and performance (Tiller, 2019). An increasing body of research indicates a significant association between respiratory muscles and running performance and athletic ability (HajGhanbari et al., 2013). During running, the respiratory muscle group needs to generate sufficient force to meet the demands of gas exchange, and robust respiratory muscles can provide better breathing efficiency and oxygen supply, thereby influencing the endurance and speed of running (Aliverti et al., 1997). The respiratory muscles refer to the group of muscles involved in the breathing process, with the diaphragm being the most vital muscle responsible for the normal execution of respiratory movements (West, 2012). The strength of the respiratory muscles plays a critical role in the breathing process and is considered an important marker of respiratory capacity and overall performance (Silva et al., 2018). Fatigue of the respiratory muscles during high-intensity or sustained exercise increases sympathetic nervous system activity and constriction of blood vessels in the exercising limbs, which can impair blood flow to the muscles, subsequently affecting athletic performance (St Croix et al., 2000; Dempsey et al., 2002). Therefore, incorporating respiratory muscle training can help delay muscle fatigue and improve athletic performance (Held and Pendergast, 2014). Among the various parameters used to assess respiratory muscle strength, Maximal Inspiratory Pressure (MIP) and Maximum Expiratory Pressure (MEP) are crucial indicators that hold significant implications for evaluating respiratory function and predicting changes in lung capacity (Pessoa et al., 2014). They are widely employed as effective measures for assessing respiratory muscle strength (Li et al., 1986) and provide a fast and non-invasive method to gauge diaphragm strength (Sachs et al., 2009). The measurement of MIP and MEP typically involves using specialized devices such as a negative pressure peak flow meter or respiratory muscle strength meter, allowing the individual being tested to measure the maximum negative pressure generated during maximum inhalation or exhalation (Schoser et al., 2017).

In recent years, with the advancement of runners’ capabilities, there has been a proliferation of research on predicting marathon performance. However, most studies have primarily focused on variables such as maximal oxygen uptake, body composition, and running mechanics (Nikolaidis and Knechtle, 2023) Surprisingly, there is a dearth of research concerning the evaluation of respiratory muscle levels in marathon runners, despite evidence suggesting that improvements in respiratory muscle function can enhance endurance performance in middle to long-distance running (Chang et al., 2021). Evaluating the respiratory muscle levels in marathon runners holds significant importance in defining the athletes’ potential speed limits and achieving optimal performance.

The aim of this study is to develop a preliminary predictive model of runner training and health management by surveying and measuring amateur marathoners. Also, a standardized test of respiratory muscle capacity will be used to collect personal information including age, gender, height, weight and related factors. We hypothesize that MIP and MEP are associated with half-marathon performance.

## 2 Objects and methods

### 2.1 Research object

Participants were recruited from the official “Olsen Exercise 2023 Beijing Olympic Forest Park Half Marathon” held at the Beijing Olympic Forest Park in 2023. Inclusion criteria were as follows: 1) registered participants of the half marathon who completed the race; 2) voluntary participation in the testing process; 3) good physical condition; 4) abstained from food intake within 3 h before the testing. Exclusion criteria included: 1) failure to complete the race; 2) engagement in high-intensity exercise or staying up late within 24 h before the testing; 3) use of medications that could affect respiratory function. After careful screening and data cleansing, a total of 233 participants’ data were selected (Table 1). These participants’ data were randomly divided into two groups: a modeling group consisting of 183 individuals and a validation group consisting of 50 individuals. There were no statistically significant differences in the basic characteristics between the two groups of participants.

**TABLE 1:** Basic Overview of Metrics for modeling group and validation group.

Group	n	Age	Gender (male/female)
modeling group	183	41.80 ± 11.73	92/91
validation group	50	40.74 ± 9.42	26/24
P		0.455	

### 2.2 Height and weight measurement

#### 2.2.1 Testing instruments

Instrumentation The Inbody720 body (Biospace, Seoul, South Korea) composition analyzer was used for the measurements.

#### 2.2.2 Test methods

Measurement Procedure Participants were instructed to stand barefoot and dry on the electrode plates of the analyzer, ensuring full contact between their feet and the electrodes. They were also asked to hold the device’s handle while maintaining contact between their fingers and the electrodes. Once the measurement was completed, the data were recorded.

### 2.3 Maximum inspiratory pressure and maximum expiratory pressure testing

#### 2.3.1 Testing instruments

Instrumentation The respiratory muscle assessment and training device (model JL-REX01F, with pressure sensors from ATS in the United States and ERS in Europe, with an error margin of 0.5 cmH2O) was used for the testing.

#### 2.3.2 Test methods

The subject sat in the front 1/2 of the chair in an upright position, cleaned the foreign matter in the nasal cavity, put on a nose clip, opened the legs stepped on the ground with shoulder width, and kept the upper body straight. The MIP test requires the subject to completely exhale to the residual volume, then wrap the mouthpiece tightly with the mouth immediately after exhaling, and inhale through the mouth for about 3 s. The test operator encourages the subject verbally. The MEP test requires the subject to inhale completely to saturation, then wrap the mouthpiece with his mouth immediately after inhaling, and exhale through the mouth for about 3 s. The test operator encourages the subject verbally. For time reasons, instead of providing a specific warm-up, subjects were allowed 2 maximal attempts per test, with approximately 60 s between each attempt. If the procedure is performed incorrectly (e.g., using the buccinator muscle), the measurement for that attempt is not recorded. During the measurement process, the same operator performs the measurement using the above-unified process.

### 2.4 Establishment of prediction equation and cross-validation

Multiple linear regression analysis was employed to establish a regression equation, with the participants’ half-marathon time as the dependent variable and their age, gender, height, weight, MEP, and MIP as independent variables in a stepwise manner. After establishing the equation, the measurement data of the 50 participants in the validation group were inputted into the regression equation for cross-validation. The predicted half-marathon time was compared with the actual measured half-marathon time, and the correlation was analyzed.

### 2.5 Statistical analysis

The collected data were double-entered into Microsoft Excel 2019, and the results were presented as mean ± standard deviation. Statistical analysis was performed using SPSS 21.0 software. The normal distribution of the data was assessed using the Kolmogorov-Smirnov test. Pearson correlation coefficient was used to evaluate the relationship between half-marathon performance and influencing factors (significance level set at *p* < 0.05 and highly significant at *p* < 0.01). The coefficient of determination (R2) was used to assess the strength of the explanatory variables on half-marathon performance. Paired t-tests were conducted to analyze the differences between the actual and predicted values, with a significance level of *p* < 0.05.

Gender was represented using dummy coding, with males assigned a value of 1 and females assigned a value of 2. Significant variables were selected as independent variables, and half-marathon performance was considered the dependent variable. Regression analysis was conducted using a stepwise approach to establish the regression equation. The regression model was evaluated through hypothesis testing, goodness of fit testing, residual analysis, multicollinearity testing, and cross-validation.

## 3 Results

### 3.1 Performance and relevant metrics of the model group in half marathon

Table 2 presents the basic characteristics of the 183 participants in the modeling group with respect to various running indicators. The results indicate that the mean Maximal Inspiratory Pressure (MIP) was 100.55 ± 30.62, Maximum Expiratory Pressure (MEP) was 101.44 ± 30.50, height was 168.28 ± 7.89, weight was 65.29 ± 10.97, and age was 42.91 ± 11.25. In terms of gender differences, males exhibited significantly higher values than females in all the measured indicators (*p* < 0.01).

**TABLE 2:** Basic overview of metrics for the model group.

Index	Male (*n* = 92)	Female (*n* = 91)	Ensemble
age	42.47 ± 11.86	43.36 ± 10.65	42.91 ± 11.25
Height (cm)	173.90 ± 5.90	162.60 ± 5.10*	168.28 ± 7.89
Weight (kg)	73.15 ± 8.60	57.34 ± 6.46*	65.29 ± 10.97
MIP	113.65 ± 28.61	87.31 ± 26.76*	100.55 ± 30.62
MEP	115.65 ± 31.14	87.07 ± 22.02*	101.44.±30.50
Grades (min)	121.54 ± 7.79	135.86 ± 6.99*	128.67 ± 10.30

Note: Compared to males, *p < 0.01.

### 3.2 Correlation analysis between various metrics and half marathon time in the model group

Based on the correlation analysis, as shown in Table 3, it was found that gender, height, weight, MEP, and MIP were all significantly correlated with the half marathon performance (*p* < 0.01). However, age showed no significant correlation with the half marathon performance (*p* > 0.05).

**TABLE 3:** Correlation analysis of various metrics with half marathon performance in the model group.

Indicators	r	p
Gender	0.697	*p* < 0.001
Age	0.065	*p* > 0.05
Height (cm)	−0.284	*p* < 0.001
Weight (kg)	−0.132	*p* < 0.05
MIP	−0.254	*p* < 0.005
MEP	−0.438	*p* < 0.01

### 3.3 Establishing a regression equation to predict half marathon performance

**FIGURE 1:**
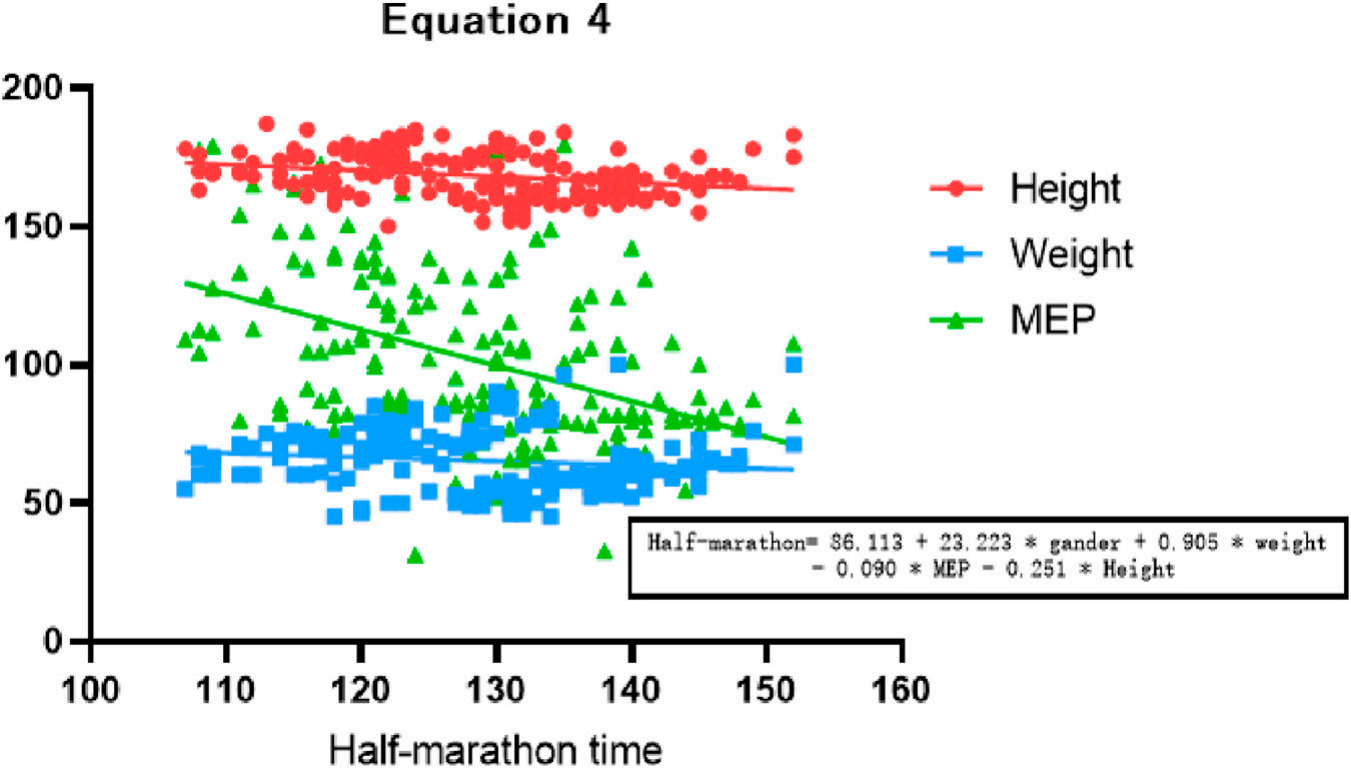
Variation of correlation coefficients in Equation 4.

The stepwise regression analysis was performed by including gender, height, weight, MIP, and MEP as predictors in the regression equation. The regression analysis results are presented in Table 4 and Table 5; Figure 1.

**TABLE 4:** Summary of regression models.

Model	R	R2	Adjusted R-square	Standard error	Durbin-watson
1	0.697^a^	0.486	0.483	7.406	
2	0.880^b^	0.774	0.772	4.924	
3	0.907^c^	0.823	0.820	4.375	
4	0.912^d^	0.832	0.828	4.266	1.939

^a^ Predictors: (Constant), Gender.

^b^ Predictors: (Constant), Gender, Weight.

^c^ Predictors: (Constant), Gender, Weight, MEP.

^d^ Predictors: (Constant), Gender, Weight, MEP, height.

**TABLE 5:** Correlation coefficients in Equation 4.

Indicators	β	Standard error	Standardized coefficients	t	P	VIF
(Constant)	86.113	11.916		7.227	0.000	
Gender	23.223	0.987	1.130	23.530	0.000	2.448
Weight (kg)	0.905	0.058	0.964	15.694	0.000	4.004
MEP	−0.090	0.012	−0.266	−7.490	0.000	1.338
Height (cm)	−0.251	0.078	−0.192	−3.194	0.002	3.837

Based on the regression results, the following equations can be derived to estimate the performance of amateur marathon runners:

Equation 1: Half-marathon time = 107.219 + 14.325 * gender.

Equation 2: Half-marathon time = 42.499 + 25.821 * gender (male = 1, female = 2) + 0.728 * weight.

Equation 3: Half-marathon time = 50.238 + 24.193 * gender (male = 1, female = 2) + 0.779 * weight—0.086 * MEP.

Equation 4: Half-marathon time = 86.113 + 23.223 * gender (male = 1, female = 2) + 0.905 * weight—0.090 * MEP - 0.251 * height.

Based on the results in Table 4:

Model 1 has a correlation coefficient (R) of 0.697, indicating a moderate positive correlation between half-marathon time and gender. The coefficient of determination (R-squared) is 0.486, suggesting that this model can explain 48.6% of the variance in half-marathon time. The adjusted R-squared is 0.483, indicating a slight improvement in the model’s explanatory power after considering the number of independent variables and sample size. The standard error is 7.406, representing the average standard deviation of the prediction errors.

Model 2 has a correlation coefficient (R) of 0.880, indicating a strong positive correlation between half-marathon time and gender and weight. The coefficient of determination (R-squared) is 0.774, suggesting that this model can explain 77.4% of the variance in half-marathon time. The adjusted R-squared is 0.772, indicating a slight improvement in the model’s explanatory power after considering the number of independent variables and sample size. The standard error is 4.924, slightly higher than in Model 1, indicating a slightly increased average standard deviation of the prediction errors.

Model 3 has a correlation coefficient (R) of 0.907, indicating a strong positive correlation between half-marathon time and gender, weight, and MEP. The coefficient of determination (R-squared) is 0.823, suggesting that this model can explain 82.3% of the variance in half-marathon time. The adjusted R-squared is 0.820, indicating a slight improvement in the model’s explanatory power after considering the number of independent variables and sample size. The standard error is 4.378, slightly lower than in Model 2, indicating a slightly reduced average standard deviation of the prediction errors.

Model 4 has a correlation coefficient (R) of 0.912, indicating a strong positive correlation between half-marathon time and gender, weight, MEP, and height. The coefficient of determination (R-squared) is 0.832, suggesting that this model can explain 83.2% of the variance in half-marathon time. The adjusted R-squared is 0.828, indicating a slight improvement in the model’s explanatory power after considering the number of independent variables and sample size. The standard error is 4.266, slightly higher than in Model 3, indicating a slightly reduced average standard deviation of the prediction errors. The Durbin-Watson statistic is 1.939, which is close to 2, indicating that the errors in this model are relatively independent.

In conclusion, Model 4 exhibits a strong explanatory power with high R-squared and adjusted R-squared values. It takes into account the influence of gender, weight, MEP, and height variables. The small standard error suggests a good fit for the model. The Durbin-Watson statistic indicates relatively independent error terms in the model. Overall, the results show that factors such as gender, weight, MEP, and height significantly influence half-marathon performance. These models have relatively strong explanatory power and small standard errors, providing valuable insights for further research.

### 3.4 Backward elimination test

Based on the results in Table 6, a validity test was conducted to compare the actual half-marathon times of the validation group with the predicted half-marathon times obtained from Model 4. The paired sample *t*-test was performed to assess the differences between the two sets of data, and a correlation analysis was conducted to examine the relationship between them.

**TABLE 6:** Discrepancy and correlation between actual and predicted values of half-marathon performance.

Data	Observed	Verified	Paired-sample *t*-test		Pearson correlation test	
			t	P	r	P
	128.04 ± 9.75	128.40 ± 9.64	−0.439	0.663	0.880	0.000

**FIGURE 2:**
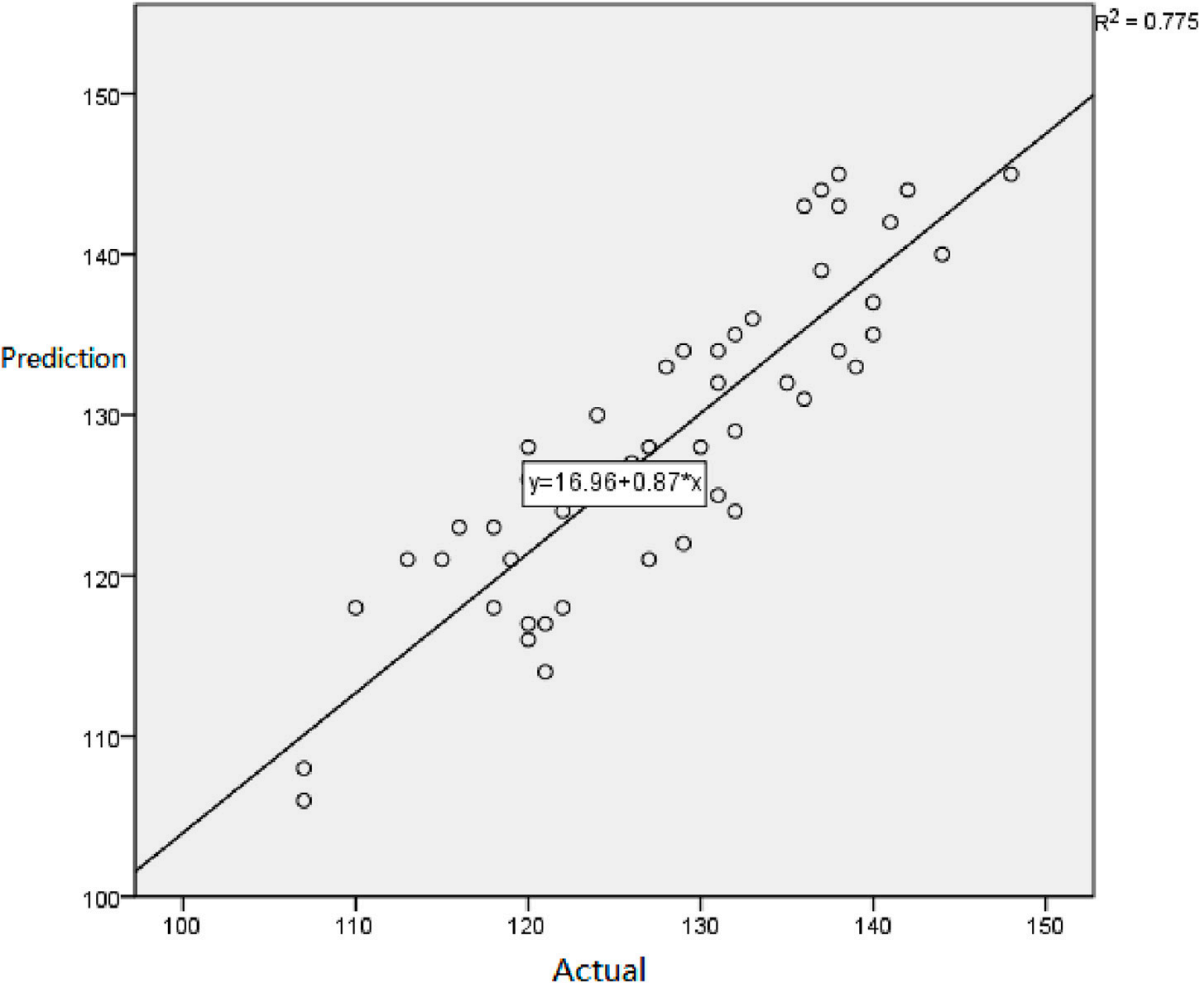
Scatter plot of predicted half-marathon and observed half-marathon values in Equation 4.

The results indicate that there is no statistically significant difference in the differences between the two sets of data (*p* > 0.05). Additionally, a significant correlation (*p* < 0.05) exists between the predicted and actual half-marathon times. Furthermore, based on Figure 2, it can be observed that there is a good linear relationship between the predicted MIP and the measured MIP.

These findings suggest that the predicted half-marathon times obtained from Model 4 are in agreement with the actual half-marathon times of the validation group. The lack of a significant difference and the presence of a significant correlation indicate the validity of the model’s predictions. Additionally, the satisfactory linear relationship between the predicted and measured MIP further supports the reliability of the model’s estimations.

## 4 Discussion

### 4.1 The scientific validity of the regression equation

Maximal inspiratory pressure (MIP) and maximal expiratory pressure (MEP) are important indicators for assessing respiratory muscle strength and aerobic capacity (Mizuno et al., 2022). In the context of running, the development of respiratory muscle strength and aerobic capacity is crucial for running performance and endurance. Higher respiratory muscle strength is closely associated with improved running performance. By enhancing respiratory muscle strength, athletes can improve gas exchange efficiency in the lungs, enhance breathing efficiency, and effectively supply oxygen to the working muscles, thus delaying the onset of fatigue (Katayama et al., 2019). The present study revealed correlations between MIP, MEP, and half-marathon performance, with MEP exhibiting the highest correlation among all variables (−0.438,*p* < 0.01). This indicates the feasibility of using respiratory muscle levels to predict half-marathon perfor mance. This finding holds particular significance for long-distance running and endurance training, as sustained oxygen supply is vital for maintaining prolonged athletic performance. Moreover, higher MIP and MEP are also associated with running speed and explosive power. Robust respiratory muscle strength enables greater respiratory flow rate and lung capacity, allowing athletes to maintain normal breathing frequency and depth during high-intensity exercise (Romer and Polkey, 2008; Kilding et al., 2010). These findings underscore the importance of respiratory muscle strength not only for endurance-related activities but also for short-distance sprints, accelerations, and explosive power training. In such activities, rapid and efficient breathing supports high-intensity muscle contractions and precise technical movements.

In this study, a stepwise regression analysis was employed to establish four regression equations for predicting half-marathon performance. Comparing the multiple correlation coefficient (R), coefficient of determination (R-square), adjusted coefficient of determination (adjusted R-square), and standard error of estimate (SEE) among the established regression equations, it is evident from Table 4 that Equation 4 exhibited the best fit (R-square = 0.832). The regression equation for this model is half-marathon performance = 86.113 + 23.223 * gender (male = 1, female = 2) + 0.905 * weight—0.090 * MEP—0.251 * height. Validation of the variables in this equation against the validation dataset revealed no significant differences between the two groups (t = −0.439, *p* > 0.05), yet a significant correlation was observed (r = 0.880, *p* < 0.01).

In the subsequent steps of building the regression equation, gender entered the model in the first step, consistent with the prediction results of the other regression equations. Gender was found to have a significant influence on half-marathon performance. In the second step, weight entered the model as the second independent variable. This could be attributed to the fact that half-marathon runners, as amateur enthusiasts without professional training, exhibit variations in body weight, which is indicative of their athletic abilities. The negative correlation between weight and half-marathon performance aligns with common understanding, as individuals who engage in regular physical activity, particularly in long-distance running, tend to have lower body weight compared to their counterparts of the same age group. In the third step, MEP entered the model as the third variable. This parameter demonstrated a moderate correlation with half-marathon performance (−0.438, *p* < 0.01). Previous research has also demonstrated that improvements in respiratory muscle strength can enhance performance in various exercise modalities, including running, cycling, swimming, and rowing (Shei, 2018).

### 4.2 The value of assessing respiratory muscle strength in half-marathon runners

Half-marathon performance is influenced by multiple factors. Numerous scholars, both domestically and internationally, have established different prediction models based on the underlying mechanisms and influencing factors of half-marathon performance. These models primarily focus on variables such as weekly running distance, weekly running frequency, and maximal oxygen uptake, often employing multiple linear regression as the primary analytical approach (Doherty et al., 2020). However, the incorporation of respiratory muscle strength, represented by maximal inspiratory pressure (MIP) and maximal expiratory pressure (MEP), in regression equations has been rarely explored.

Additionally, higher MIP and MEP levels are associated with running speed and explosive power. Robust respiratory muscle strength enables greater respiratory flow and lung capacity, allowing athletes to maintain normal breathing frequency and depth even at higher exercise intensities (Romer and Polkey, 2008; Kilding et al., 2010).This is especially significant for short-distance running, sprinting, and explosive training, as rapid and efficient respiration supports high-intensity muscle contractions and facilitates precise execution of technical movements.

Previous studies on respiratory muscles have primarily focused on patients and non-exercising healthy individuals. As early as 2000, researchers investigated the lower limit of normal reference values for maximal inspiratory pressure (MIP) in healthy adults aged 18–82 years with normal lung function. These reference values were intended for use in pulmonary function laboratories for both young and elderly patients (Hautmann et al., 2000). In healthy young Indonesian adults, a rapid and convenient measurement of chest expansion was found to be useful for screening respiratory muscle strength in patients (Gopalakrishna et al., 2011). A study examining maximal respiratory pressures in healthy Brazilian children revealed that boys had higher values than girls, and respiratory pressure increased with age. Age and gender were included in the formula for maximal inspiratory pressure, while age and body weight (for boys) were included in the formula for maximal expiratory pressure (Marcelino et al., 2022). Furthermore, several researchers have explored the correlation of factors other than lung function. In a 2016 study, a strong correlation (r = 0.76) was found between MIP and handgrip strength in healthy young and middle-aged individuals. A multiple linear regression model was established, suggesting that this indirect assessment could aid in evaluating the relationship between hand and inspiratory muscles, particularly the diaphragm (Raab et al., 2016). In a recent 2023 study, the relationship between inspiratory muscle strength and balance in women aged 41–80 years in the northeastern region of Brazil was investigated. The results showed that weaker inspiratory muscle strength tripled the risk of poor performance in balance testing. Early identification of individuals at risk of respiratory muscle weakness or balance impairment is crucial for preventive measures and targeted interventions (Azevedo et al., 2023). Additionally, respiratory system diseases or conditions can also affect MIP (Raab et al., 2016). Although previous studies on respiratory muscles have provided insights into healthy individuals and patients, the limited sample size and restricted generalizability, as well as the lack of standardized evaluation criteria and varying reported reference values, make them less applicable to the athletic population.

Marathon running, as a highly challenging endurance sport, places significant demands on respiratory muscle strength during prolonged and intense physical activity, playing a crucial role in maintaining respiratory function and exercise performance. Multiple studies have indicated that marathon running exerts an influence on athletes’ respiratory muscle strength. Following the completion of a marathon, athletes often experience respiratory muscle fatigue, attributed to the prolonged and intense nature of the exercise, leading to a decline in respiratory muscle strength (Loke et al., 1982). In ultra-marathon events, athletes may face even more severe respiratory muscle fatigue (Ker and Schultz, 1996). In high-altitude ultra-marathons, it has been observed that respiratory muscle fatigue is more pronounced, likely due to the hypoxic environment and increased load at high altitudes (Wuthrich et al., 2015). However, despite the changes in respiratory muscle strength and lung function following marathon running, recovery occurs relatively rapidly. Thus, the impact of marathon running on respiratory muscle strength is transient, as athletes’ respiratory muscle strength can return to normal levels within a certain recovery period (Ross et al., 2008).

Respiratory muscle strength holds significant importance for marathon performance. Following a consecutive 10-day marathon race, notable changes occur in athletes’ respiratory muscle strength and lung function. This highlights the enduring and extreme demands placed on respiratory muscle strength during marathon running, crucial for maintaining stable breathing and gas exchange (Tiller et al., 2019). Moreover, athletes’ respiratory muscle strength is closely linked to peripheral muscle strength and respiratory function. Optimal respiratory muscle strength contributes to increased lung capacity, enhanced respiratory efficiency, and provides stable respiratory support for prolonged endurance activities (Akınoğlu et al., 2019).

Due to the impact of respiratory muscle strength on marathon performance, training the respiratory muscles in marathon runners is of paramount importance. Analysis of multiple studies reveals that respiratory muscle training has positive effects on athletes’ performance. Training targeted at the respiratory muscles enhances respiratory muscle strength, increases lung capacity, and improves respiratory efficiency (HajGhanbari et al., 2013). Furthermore, respiratory muscle training improves athletes’ maximal oxygen uptake (VO2max) and respiratory threshold, positively influencing lung function. This enhances athletes’ respiratory muscle endurance and coordination, improving respiratory stability and efficiency during prolonged endurance activities (Amonette and Dupler, 2002). Training methods focusing on respiratory muscle strength, such as the use of respiratory muscle training devices and specific respiratory muscle exercises, can be incorporated into running training programs to enhance running performance and overall physical adaptation (Harms et al., 1998; Gething et al., 2004).

Marathon running has a certain impact on athletes’ respiratory muscle strength, potentially leading to respiratory muscle fatigue. Respiratory muscle strength holds significant importance for marathon performance, as it enhances lung function, improves respiratory efficiency, and provides stable respiratory support during prolonged endurance activities. Respiratory muscle training is considered an effective strategy for improving marathon performance, as it strengthens respiratory muscle strength, improves lung function, and enhances the respiratory threshold, thereby improving athletes’ respiratory stability and endurance.This study explores the association between these factors and half-marathon performance and predicts runners’ half-marathon performance through statistical analysis and regression modeling. The results of this study can provide a useful reference for scientific research and health management of running sports.

### 4.3 Limitations of the study and future directions for improvement

This study still has some limitations. The analysis of specific training activities and sports performance was not collected in the previous data collection. The research object of this study focuses on amateur marathon runners who have not received professional track and field training. Only exercise in their spare time, and do not have a fixed training time or specific training means. It is precisely for this reason that the data of these runners are beneficial to most amateurs. Still, in the future, we will further expand the amount of data and categorize them according to specific training. In addition, this study did not analyze the data separately by gender, and due to the sample size number, the DW value of the regression results due to non-open analysis is far from 2. The results are not representative, and it is speculated that the regression results will improve after the sample size is further increased. Future studies could further explore the relationship between full marathon performance and respiratory muscle strength, consider a wider sample population, and incorporate maximal inspiratory pressure (MIP) into the equation to improve its predictive value for marathon performance. In summary, increasing test density, expanding sample size, standardizing performance stratification, and collecting physiological and biomechanical data will be key to further deepening the understanding of the influencing factors and improving the accuracy of the prediction equation in future studies.

## 5 Conclusion

This study focused on 233 amateur half-marathon runners as participants and analyzed the correlations between respiratory muscle strength and various variables with half-marathon performance. The results indicated significant correlations (*p* < 0.05) between gender, weight, height, Maximum Expiratory Pressure (MEP), and half-marathon performance. Consequently, these variables were selected as independent variables in the regression equation. The regression equation was as follows: Half-Marathon Performance = 86.113 + 23.223 Gender (Male = 1, Female = 2) + 0.905 Weight—0.090MEP—0.251Height. Furthermore, the reliability and validity of this equation were tested and confirmed. Therefore, using gender, height, weight, and MEP as predictors yields a reasonably accurate estimation of half-marathon performance. The findings of this study provide valuable insights for scientific research and health management in the field of running.

## Data Availability

The original contributions presented in the study are included in the article/Supplementary material, further inquiries can be directed to the corresponding author.
