# Corrigendum: A preliminary exploration of the regression equation for performance in amateur half-marathon runners: a perspective based on respiratory muscle function

**DOI:** 10.3389/fphys.2024.1420347

**Published:** 2024-05-24

**Authors:** Houyuan Zhu, Xiaowei Han, Guoqing Miao, Qi Yan

**Affiliations:** ^1^ China Institute of Sport Science, Beijing, China; ^2^ School of Physical Education, Hebei Normal University, Shijiazhuang, Hebei, China

**Keywords:** half marathon, maximum expiratory pressure, multiple linear regression, respiratory muscle, running

In the published article, there were some errors in the authors’ **Affiliations**. Instead of “School of Physical Education, Hebei Normal University, Shijiazhuang, Hebei, China”, affiliation 1 should be “China Institute of Sport Science, Beijing, China”. Instead of “China Institute of Sport Science, Beijing, China”, affiliation 2 should be “School of Physical Education, Hebei Normal University, Shijiazhuang, Hebei, China”.

In the published article, there was an error regarding the affiliation for Houyuan Zhu. As well as having affiliation 1, they should also have affiliation 2.

The authors apologize for these errors and state that this does not change the scientific conclusions of the article in any way. The original article has been updated.

